# 3,3’-Diindolylmethane and its derivatives: nature-inspired strategies tackling drug resistant tumors by regulation of signal transduction, transcription factors and microRNAs

**DOI:** 10.20517/cdr.2020.53

**Published:** 2020-10-12

**Authors:** Bernhard Biersack

**Affiliations:** Organic Chemistry 1, University of Bayreuth, Bayreuth 95440, Germany.

**Keywords:** Indole, 3, 3’-diindolylmethane, drug resistance, anticancer drugs, microRNAs

## Abstract

Indoles of cruciferous vegetables are promising anti-tumor agents. Studies with indole-3-carbinol and its dimeric product, 3,3’-diindolylmethane (DIM), suggest that these compounds have the ability to deregulate multiple cellular signaling pathways that are essential for tumor growth and spread. These natural compounds are also effective modulators of transcription factors and non-coding RNAs. These effects explain their ability to inhibit tumor spread and to overcome drug resistance. In this work, pertinent literature on the effects of DIM and its synthetic derivatives on resistant tumors and resistance mechanisms in tumors is highlighted.

## Introduction

**Figure 1:**
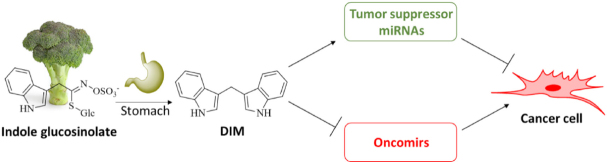
Natural indole glucosinolates form 3,3’-diindolylmethane (DIM) in the stomach, which suppresses tumor growth and overcomes tumor resistance by induction of tumor suppressor miRNAs and suppression of oncomirs

Cancer is one of the most serious human health concerns. The current treatments of cancer comprise of surgery, radiation therapy, chemotherapy with classical cytotoxic drugs and/or targeted drugs (e.g., protein kinase inhibitors), endocrine therapy, and immunotherapy^[1-3]^. However, efficient cancer treatment with chemotherapeutics is hampered by drug resistance. The formation of tumor resistance is complex and based on intrinsic and acquired resistance mechanisms which include increased efflux of chemotherapeutics (e.g., by ABC transporters), increased DNA repair, mutation or alteration of drug targets, epigenetic mechanisms such as epigenetic regulation of gene expression and/or of protein drug targets, induction of senescence, factors in the tumor microenvironment, and epithelial-to-mesenchymal transition^[4,5]^. In order to overcome these resistance factors, thorough knowledge of these mechanisms is necessary in addition to the identification of new drugs. Nature-derived indole compounds have shown great potential as anticancer agents, and indole alkaloid drugs such as vincristine and vinblastine are well established for the treatment of tumor diseases since many years^[6,7]^. The indole-based protein kinase inhibitors sunitinib (approved for the treatment of metastatic renal cell carcinoma) and enzastaurin were developed based on the natural lead indole derivative staurosporin^[8-10]^. Indoles are also prominent dietary compounds, and indole alkaloid derivatives of *Brassica* species such as indole glucosinolates, indole-3-carbinol (I3C), and 3,3’-diindolylmethane (DIM) display distinct anticancer activities based on apoptosis induction as well as suppression of phosphatidyl-inositol-3-kinase(PI3K)/Akt and nuclear factor κB (NF-κB) signaling [Figure 1]^[11-13]^. Long ago, Cato the Elder recommended cabbage leafs for the treatment of cancerous ulcers and statistics now show that populations with increased consumption of cruciferous vegetables showed lower cancer incidences^[13-15]^. The natural indole glucosinolates decompose into I3C and form DIM in the stomach upon consumption. However, the bioavailability of DIM is poor and formulations are often necessary for *in vivo* tests^[11,16]^. Synthetic derivatives of DIM have been prepared by various synthetic methods^[17,18]^. Several DIM derivatives have revealed high activities against cancer cells^[7,19]^. The current state of the anticancer activities of DIM and its synthetic derivatives is presented in this review with a focus on cancer drug resistance, tumor growth inhibition, and new insights concerning their effects on signaling pathways and transcription factors.

## DIM and cancer drug resistance - the role of micro-RNAs

The general anticancer properties of DIM based on mechanisms such as apoptosis induction, blocking of NF-κB, Akt and Wnt signaling, effects on PI3K/Akt/mTOR signaling and aryl hydrocarbon receptor (Ahr) signaling as well as antioxidant properties have already been reviewed^[7,11,20,21]^. DIM has also been shown to prevent liver cancer formation and protect hepatic tissues by suppression of TGF-β, Smad2/Smad3, AP-1, and NADPH^[22]^. Formulated DIM (BR-DIM or B-DIM = BioResponse-DIM) was well tolerated by healthy individuals and reached clinical trials for the treatment of prostate cancer and breast cancer^[11,23,24]^. In non-metastatic castrate-resistant prostate cancer patients, BR-DIM was quickly absorbed (within 2 to 4 h) and led to prostate-specific antigen (PSA) reduction, however, eventual progression was observed^[25]^. Another study of BR-DIM in prostatectomy patients with prostate cancer showed that BR-DIM was well tolerated, and DIM was detected in prostate biopsies and blood plasma. Interestingly, 96% of the treated patients showed exclusion of the androgen receptor from the cell nucleus, and 71% of the treated patients exhibited reduced PSA levels^[26]^. Likewise, BR-DIM was well tolerated in prostate cancer patients undergoing prostatectomy, DIM was detected in the blood plasma and the prostate tissue, and it increased the 2-hydroxyestrone (2-OHEI) to 16-hydroxyestrone (16α-OHEI) ratio as well as the levels of metabolic enzymes including CYP1A1 and CYP2B6^[27]^. Breast cancer patients who received BR-DIM together with tamoxifen showed increased serum androgen hormone binding globulin levels and 2/16α-OHEI ratio^[28]^.

Research on the effects of DIM on microRNAs (miRNAs), which regulate the expression of cancer cell targets of oncologic relevance, found substantial results. There are both tumor suppressor miRNAs and oncogenic miRNAs (oncomirs), which underline the role of miRNAs as a tool for the circumvention of drug resistance by DIM [Figure 1]^[29]^. Hence, a concise summary of the effects of DIM on miRNA expression in various tumor models is given in Table 1.

**Table 1:** Effects of DIM on miRNA expression in tumors

Tumor	miRNA	miRNA regulation	Targets
Prostate cancer	let-7	up	EZH2
	miR-34a	up	Notch-1, AR
	miR-150-5p	up	Ahr
	miR-92a	down	RANKL
Breast cancer	miR-200	up	FoxM1
	miR-212/132 cluster	up	Sox4
	miR-21	up	Cdc25A
Pancreatic cancer	let-7b/c/d/e	up	ZEB-1, E-cadherin
	miR-200b/c	up	ZEB-1, E-cadherin
	miR-146a	up	MTA2, NF-κB, IRAK1, EGFR
	miR-221	down	PTEN, PUMA, p27, p57

DIM: 3,3’-diindolylmethane; NF-κB: nuclear factor κB; EGFR: endothelial growth factor receptor

Various tumor suppressing miRNAs were upregulated by DIM or formulated BR-DIM [Table 1]. For instance, BR-DIM inhibited prostate tumor growth by upregulation of the tumor suppressor miRNA let-7, which led to the down-regulation of EZH2, a histone-lysine *N*-methyltransferase target of let-7 in prostate cancer patients^[30]^. In castrate-resistant prostate cancer cells, DIM induced the expression of miR-34a which was accompanied by decreased self-renewal of prostate cancer cells and suppression of Notch-1 and androgen receptor signaling^[31]^. DIM also promoted an Ahr-mediated expression of the tumor suppressor miR-150-5p in prostate cancer cells^[32]^. A modulation of the expression of miRNAs was also observed in breast cancer models. When combined with herceptin, DIM upregulated the expression of the tumor suppressor miR-200, associated with apoptosis induction and FoxM1 suppression in HER-2/neu expressing SKBR3 and MDA-MB-468 breast carcinoma cells^[33]^. An Ahr-mediated induction of miR-212/132 cluster expression was also promoted by DIM in the drug-resistant breast cancer models T47D and MDA-MB-231, which led to downregulation of the pro-metastatic SRTY-related HMG-box 4 (Sox4) protein. These effects were observed *in vitro* and *in vivo*, and aside from reduced tumor growth, the formation of metastases was inhibited as well^[34]^. In gemcitabine-resistant pancreatic cancer cells (MiaPaCa-2), DIM upregulated the expression of E-cadherin and suppressed zinc finger E-box binding homeobox 1 (ZEB1), vimentin, and slug, leading to a reversal of epithelial-to-mesenchymal transition^[35]^. These effects were correlated with an upregulation of the tumor suppressors let-7b, let-7c, let-7d, let-7e, and miR-200b/c by DIM. In addition, DIM inhibited cancer cell invasion by suppression of metastasis-associated protein 2 (MTA2), NF-κB, interleukin 1 receptor-associated kinase 1 (IRAK1), and epidermal growth factor receptor (EGFR) based on upregulation of miR-146a^[36]^.

The suppressing effects of DIM on the expression of oncogenic miRNAs/oncomirs are also shown in Table 1. DIM blocked prostate cancer bone metastasis formation by suppression of receptor activator of nuclear factor-κB ligand (RANKL) signaling and downregulation of miR-92a^[37]^. The oncomir miR-221 was suppressed by DIM in pancreatic cancer cells (MiaPaCa-2), and the upregulation of miR-221 targets such as phosphatase and tensin homolog deleted on chromosome (PTEN), p53 upregulated modulator of apoptosis (PUMA), and the cyclin-dependent kinase (CDK) inhibitors p27 and p57 was observed^[38]^. MiR-21 is a well described oncomir; however, DIM enhanced the expression of miR-21 in order to inhibit cancer cell growth and to promote Cdc25A degradation in breast cancer cells^[39]^. Nevertheless, molecular docking and molecular dynamics simulation calculations revealed that DIM can form interactions with the bases of the pre-miR-21, the precursor of miR-21, and the structure of DIM can be applied for the development of small molecule antagonists of miR-21^[40]^.

## Synthetic DIM derivatives and cancer drug resistance

**Figure 2:**
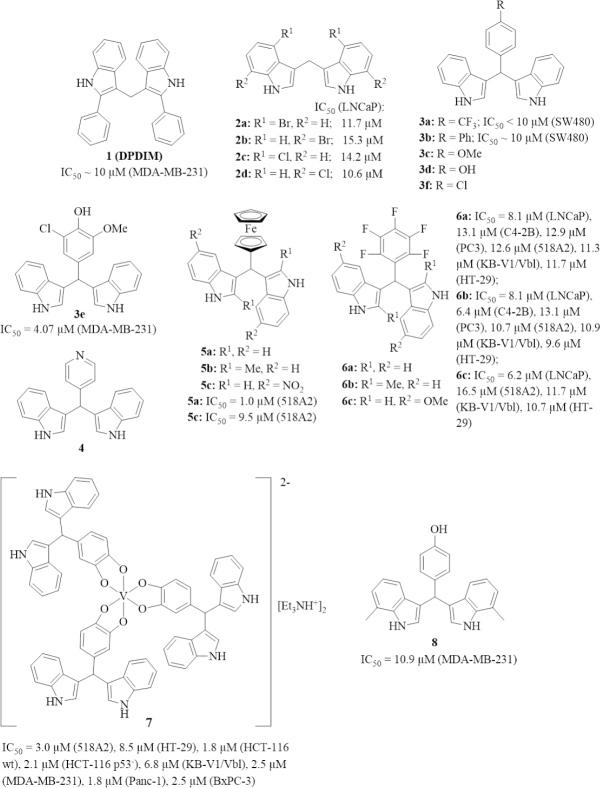
Structures of 3,3’-diindolylmethane (DIM) derivatives (halo-, phenyl, and ferrocenyl derivatives) with anti-tumor activities associated with circumvention of cancer resistance. Cell lines (in parentheses): MDA-MB-231 breast cancer, SW480 colon cancer, 518A2 melanoma, LNCaP prostate cancer, C4-2B prostate cancer, PC3 prostate cancer, KB-V1/Vbl cervix cancer, HT-29 colon cancer, HCT-116 colon cancer, Panc-1 pancreas cancer, and BxPC-3 pancreas cancer cells

Synthetic derivatives of DIM were prepared in order to obtain more anti-tumor active compounds [Figure 2]. A simple phenyl-substituted DIM derivative, 2,2’-diphenyl-3,3’-diindolylmethane 1 (DPDIM), inhibited the growth of MDA-MB-231 triple-negative breast cancer (TNBC) cells (IC_50_ ca. 10 µmol/L; triple-negative refers to breast tumor cells lacking ER/estrogen receptor and PR/progesterone receptor, and no HER-2 overexpression). The bioavailability of 1 was high enough to reduce DMBA-induced breast tumor growth in rats at doses of 5 mg/kg. The anti-tumor activity of 1 is based on the induction of apoptosis via suppression of EGFR signaling. Docking studies revealed that 1 can bind to the ATP-binding site of EGFR^[41]^. Halogen-substituted DIM derivatives 2a-d were prepared and tested for their activity against prostate cancer. All four compounds inhibited the growth of LNCaP prostate cancer cells in the presence of dihydrotestosterone (DHT) and suppressed PSA release. Compounds 2a and 2b also suppressed androgen receptor expression in the prostate cancer cells^[42]^. In addition, compounds 2a-d induced apoptosis and necrosis in androgen-dependent and androgen-independent prostate cancer cells by activation of caspases-3, -8, and -9, and induced expression of Fas, FasL, DR4, and DR5^[43]^. Compounds 2a and 2d also induced autophagy in prostate cancer cells by activation of AMP-activated kinase (AMPK) signaling and astrocyte elevated gene 1 (AEG-1)^[44]^.

Aryl substituents at the bridging methylene group of DIM can be easily introduced by reaction of indole with the corresponding aryl aldehyde. Safe *et al*.^[19]^ have prepared a series of such anti-tumor active DIM derivatives [Figure 2]. For instance, para-substituted phenyl derivatives such as 3a and 3b were identified as PPARγ activators with distinct activity against SW480 colon cancer cells. But in contrast to I3C and DIM, these synthetic compounds activated Akt. In addition, 3b (20 mg/kg) inhibited the growth of SW480 tumors *in vivo* and the tumor remnants showed upregulated caspase-3 and non-steroidal anti-inflammatory drug activated gene 1 (NAG-1)^[45]^. The anisyl analog 3c activated the orphan nuclear receptor NR4A1 (Nur77, TR3) in pancreatic tumors and inhibited the growth L3.6pL pancreatic tumors in mice at a dose of 25 mg/kg/day by NR4A1-dependent induction of apoptosis. Hence, both 3b and 3c have sufficient bioavailability in order to exert visible tumor growth inhibition and *in vivo* effects. The expression of several growth inhibitory and pro-apoptotic factors such as activating transcription factor 3(ATF3 ), p21, tumor necrosis factor-related apoptosis inducing ligand (TRAIL), and Fas ligand was induced by 3c^[46]^. In contrast to that, the phenol analog 3d was identified as an NR4A1 antagonist. Because phenol 3d is prone to metabolism, the 3-chloro-5-methoxyphenyl derivative 3e was prepared, which was distinctly more active than 3d against MDA-MB-231 breast tumors *in vitro* and *in vivo*. Compound 3e suppressed NR4A1 activity, induced p21 expression, and inhibited MDA-MB-231 xenograft tumor growth at low doses of 2 mg/kg/day, indicating a sufficient bioavailability of 3e^[47]^. The close 4-chlorophenyl analog 3f selectively activated NR4A2 (Nurr1) in pancreatic cancer cells and had only marginal effects on NR4A1 and NR4A3 activity^[48]^. In addition, 3f was no substrate of the cancer drug resistance associated drug efflux transporter Pgp/MDR1/ABCB1 (permeability glycoprotein, multidrug resistance protein 1, ATP-binding cassette sub-family B member 1)^[49]^.

The 4-pyridyl compound 4 showed stronger activation of the orphan receptor chicken ovalbumin upstream promoter-transcription factor I (COUP-TFI) in breast cancer cells than its 3-pyridyl and 2-pyridyl analogs at doses of 5, 10, and 15 µmol/L in a luciferase gene reporter assay, and 4 also induced early growth response 1 (Egr-1) activation^[50]^. There is conflicting data on how far the activation of COUP-TFs breast cancer cells can contribute to an anti-tumor treatment. On the one hand, COUP-TF was reported to suppress estrogen-induced gene expression while on the other hand another report described a proliferation promoting activity of COUP-TFI in MCF-7 breast cancer cells^[51,52]^. Hence, more research is necessary in order to find out whether 4 can be a suitable drug candidate for the treatment of breast cancer.

In continuation of this promising synthetic strategy developed by Safe *et al*.^[19]^, organometallic ferrocene derivatives 5a-c of DIM were synthesized by our groups, which were tested against various tumor cell lines [Figure 2]. 5a (IC_50_ = 6.9 µmol/L) and 5b (IC_50_ = 9.8 µmol/L) were much more active than DIM (IC_50_ = 32.1 µmol/L) against MDA-MB-231 breast cancer cells. Further to this, compounds 5a-c reached IC_50_ values below 5 µmol/L when tested against BxPC-3 pancreas cancer cells where DIM was only moderately active (IC_50_ = 25.2 µmol/L). Against DIM-resistant (IC_50_ > 100 µmol/L for DIM) tumor cell lines 518A2 melanoma, KB-V1/Vbl cervix carcinoma (i.e., vinblastine-resistant cells) and HT-29 colon carcinoma, compound 5a (DIMFc) was active at doses below 10 µmol/L (IC_50_ = 1.0 µmol/L for 518A2, 3.0 µmol/L for KB-V1/Vbl, 6.3 µmol/L for HT-29) while 5c was slightly less active (IC_50_ = 9.5 µmol/L for 518A2, 12.6 µmol/L for KB-V1/Vbl, 15.6 µmol/L for HT-29) than 5a against these tumor cells^[53]^. Ferrocene 5a overcame DIM resistance in certain tumors; thus, further development of this compound appeared promising. More recently, the pentafluorophenyl derivatives 6a-c (DIMPF series of compounds) also showed distinct activities against MDA-MB-23 breast cancer and BxPC-3 pancreas cancer cells as well. In addition, 6a-c showed significant activity against androgen-dependent (LNCaP) and androgen-independent (C4-2B, PC-3) prostate cancer cells (IC_50_ = 6.2-13.1 µmol/L), and they were able to overcome DIM resistance of 518A2 melanoma, KB-V1/Vbl cervix carcinoma, and HT-29 colon carcinoma cells (IC_50_ = 9.6-16.5 µmol/L)^[54]^.

A remarkable non-oxido vanadium (IV) complex 7 with three 3,4-dihydroxyphenyl substituted DIM ligands was also investigated for its anti-tumor activities. Complex 7 exhibited high growth inhibitory activity (IC_50_ = 1.8-3.0 µmol/L) against 518A2 melanoma, HCT-116 colon carcinoma (both p53-wildtype and p53-negative cells), triple-negative MDA-MB-231 breast cancer, and Panc-1 and BxPC-3 pancreas cancer cells. Additionally, complex 7 showed much higher activity than its metal-free catechol-modified DIM ligand against HT-29 colon cancer (IC_50_ = 8.5 µmol/L for 7, 48.9 µmol/L for the ligand) and KB-V1-Vbl cervix carcinoma cells (IC_50_ = 6.8 µmol/L for 7, 43.7 µmol/L for the ligand). The mechanisms of complex 7 were studied, and 7 interacted with DNA as shown in ethidium bromide assays, caused mitochondrial damage, produced reactive oxygen species (ROS), and led to G_2_/M cell cycle arrest in 518A2 melanoma cells^[55]^.

The 4-hydroxyphenyl derivative 8 (phemindole) is another DIM derivative with distinct and selective growth inhibitory activity against MDA-MB-231 breast cancer cells (IC_50_ = 10.9 µmol/L) and 8 was much more active than DIM (IC_50_ = 72.3 µmol/L) against these cancer cells. Compound 8 induced mitochondria-based apoptosis and ROS formation as well as endoplasmic reticulum (ER) stress by suppression of stromal interacting molecule 1 (STIM1) and store operated calcium entry (SOCE). *In vivo* experiments with 4T1 breast tumor bearing mice showed that 8 at doses of 10 mg/kg and 15 mg/kg (intravenously) was able to inhibit breast tumor growth significantly, which indicates that 8 possesses a reasonable bioavailability^[56]^.

Various formulations were described for Safe and colleagues’ para-substituted DIM-P (phenyl-DIM) derivatives. A spray-dried enteric coated self-emulsified drug delivery system (Spray BIO-Max) was applied for the formulation of DIM-Ps, which led to increased oral bioavailability and to enhanced lung tumor weight /volume reduction in mice with H1650 metastatic tumors (20%-25% greater reduction) or A549 orthotopic tumors (25%-30% greater reduction) than DIM-P solution alone^[57]^. Similarly, nano-structured lipid carrier (NLC) formulations and self-emulsifying drug (SED) delivery formulations of DIM derivatives revealed promising anticancer results in TNBC (MDA-MB-231) and lung tumor (H1650) models aside improved oral bioavailability^[58,59]^.

**Figure 3:**
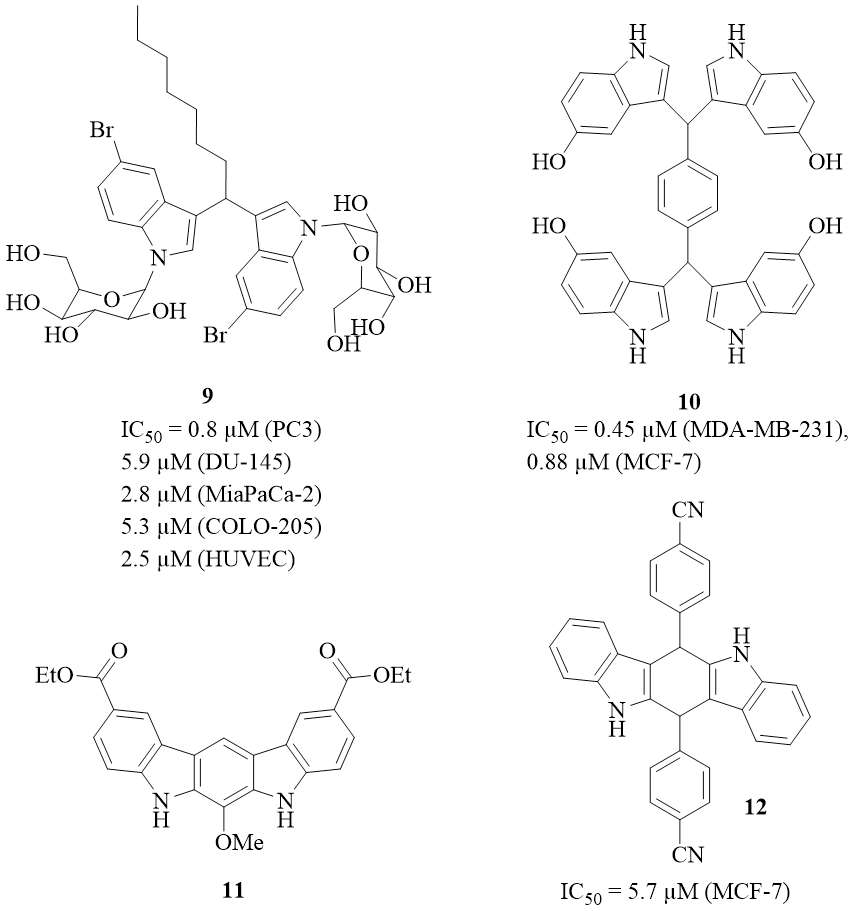
Structures of anti-tumor active 3,3’-diindolylmethane (DIM) derivatives (glycosides, tetraindole, and stabilized derivatives). Cell lines: A549 lung cancer, HeLa cervix cancer, MCF-7 breast cancer, PC3 prostate cancer, DU-145 prostate cancer, MiaPaCa-2 pancreas cancer, COLO-205 colon cancer, MDA-MB-231 breast cancer, SKOV-3 ovarian cancer, and HUVEC endothelial cells

A series of *N*-glycosides of DIM was prepared and tested for anti-tumor activity [Figure 3]. Compound 9 (NGD16) exhibited the highest tumor cell growth inhibitory activity of this series (IC_50_ = 1.3 µmol/L for A549 lung, 0.3 µmol/L for HeLa cervix, and 0.9 µmol/L for MCF-7 breast cancer cells), and induced apoptosis by upregulation of pro-apoptotic Par-4 (prostate apoptosis response 4) accompanied by suppression of Bcl-2 and GRP78 (glucose regulated protein 78 kDa). In addition, 9 inhibited the migration of HeLa cells and arrested the cell cycle in the G1 phase^[60]^. Compound 9 also inhibited the growth of moderately aggressive PC3 (IC_50_ = 0.8 µmol/L) and DU-145 prostate cancer cells (IC_50_ = 5.9 µmol/L), MiaPaCa-2 pancreas cancer cells (IC_50_ = 2.8 µmol/L), COLO-205 colon cancer cells (IC_50_ = 5.3 µmol/L), and HUVECS (IC_50_ = 2.5 µmol/L). The high activity against HUVECs suggested an anti-angiogenic mode of action for 9. Indeed, compound 9 inhibited angiogenesis by suppression of GRP78, VEGFR2 (vascular endothelial growth factor receptor 2), and matrix metalloproteinase-9 (MMP-9) expression, and cancer cells treated with 9 showed a translocation of GRP78 to the cell membrane and interaction of GRP78 with tissue inhibitor of metalloproteinase-1 (TIMP-1)^[61]^. Since Par-4 is a known inhibitor of tumor invasion, epithelial-to-mesenchymal transition, and mesenchymal markers, compound 9 was also studied for its inhibitory effects on the epithelial-to-mesenchymal transition. In pancreas cancer cells, 9 induced Par-4 leading to the suppression of vimentin, Twist-1, and Sox2. Additionally, ALK2/Smad4 signaling was induced by 9 via Par-4 activation. Hence, compound 9 appeared to be a promising drug candidate for the treatment and prevention of metastatic pancreas cancer^[62]^.

The tetraindole 10 (SK228) prepared from terephthalaldehyde and 5-hydroxyindole showed higher activity against triple-negative MDA-MB-231 breast cancer cells (IC_50_ = 0.45 µmol/L) than against hormone-sensitive MCF-7 breast cancer cells (IC_50_ = 0.88 µmol/L)^[63]^. Compound 10 led to G_2_ cell cycle arrest by upregulation of cyclin B1 expression and phosphor-cdc-2 in the breast cancer cells. The stable DIM derivative 11 (SR13668) conserved the Akt suppressing properties of I3C and DIM in PC3 prostate cancer cells and inhibited the growth of triple-negative MDA-MB-231 breast tumors, androgen-independent PC-3 prostate tumors, and drug-resistant SKOV-3 ovarian tumors *in vivo* indicating a reasonable bioavailability of 11^[64]^. Tetrahydroindolocarbazoles are further promising structural analogs of DIM, and compound 12 showed activity against MCF-7 breast cancer cells (IC_50_ = 5.7 µmol/L). 12 inhibited the tumor-promoting factor uPA (urokinase plasminogen activator) which is overexpressed in various cancers including breast cancer^[65]^.

## Conclusion

**Figure 4:**
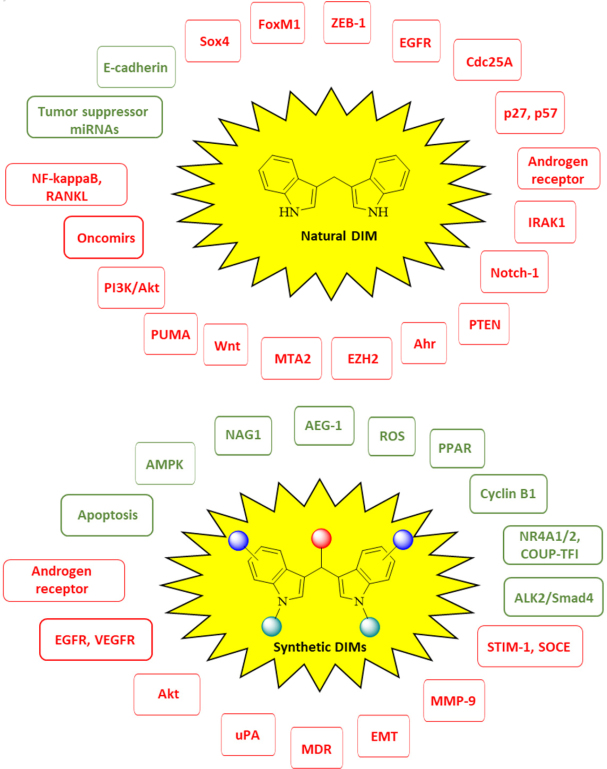
Targets of natural 3,3’-diindolylmethane (DIM) and synthetic DIM derivatives (green = activated targets, red = suppressed/inhibited targets)

There is growing evidence that natural DIM and synthetic DIM derivatives are useful tools in order to overcome cancer drug resistance. These drug candidates use diverse and manifold mechanisms in order to break resistance or to re-sensitize resistant tumors for approved anticancer drugs [Figure 4]. Certain DIM derivatives were even active against DIM resistant tumor cell lines, others showed promising *in vivo* activities against metastatic and drug-resistant tumor models. In addition, valuable formulation strategies were developed in order to increase the bioavailability of DIM and of some of its derivatives and to enhance their *in vivo* anti-tumor activity. These strategies appear also to be useful for the formulation of other existing or future DIM derivatives with similar bioavailability problems.

## Declarations

### Copyright

© The Author(s) 2020.
